# Mapping of Organ Radiation Dose Distribution in Abdominal X-ray Imaging Using Optically Stimulated Luminescent Dosimeters (OSLDs)

**DOI:** 10.7759/cureus.85701

**Published:** 2025-06-10

**Authors:** Inayatullah Shah Sayed, Nur Izzatie Arissya Rohayzaad, Nur Nabilah Muhamad Jamil, Azia Alina Kamzaiman

**Affiliations:** 1 Department of Diagnostic Imaging and Radiotherapy, Kulliyyah of Allied Health Sciences, International Islamic University Malaysia, Kuantan Campus, Kuantan, MYS

**Keywords:** abdominal radiography, anode heel effect, esd, non-uniform radiation intensity, patient radiation safety, radiation dosimetry

## Abstract

Introduction: X-ray imaging units often exhibit non-uniform radiation intensity across the imaging field, making accurate dose measurements challenging. This variability can impact patient safety and hinder the optimization of medical imaging procedures. Our study aims to map radiation doses in abdominal organs using optically stimulated luminescent dosimeters (OSLDs) to enhance dose accuracy and improve the quality of patient care in diagnostic imaging.

Materials and methods: A Kyoto Kagaku phantom (Kyoto, Japan) was placed supine on the table Bucky in a posteroanterior (PA) orientation, with a source-to-image receptor distance (SID) of 100 cm. Exposures were taken at three different kilovolt peak (kVp) settings (64.5, 70, and 75) with corresponding milliampere-seconds (mAs) values of 15, 20, and 25, respectively, using both anode- and cathode-oriented beams. Multiple nanoDot OSLDs (Landauer, Glenwood, IL, US) were positioned on the phantom’s surface to measure the entrance surface dose (ESD) at anatomical locations corresponding to the liver, kidneys, and spleen.

Results: ESD measurements revealed a non-uniform radiation distribution across abdominal organs. Organ doses increased with rising kVp and mAs, reflecting the influence of beam energy and exposure settings. With anode-facing beam orientation, liver doses ranged from 0.47 to 2.97 mGy, with higher values in central liver regions and right kidney segments. ESD to the spleen increased from 0.63 to 1.94 mGy. Under cathode-oriented conditions, liver doses ranged from 0.88 to 3.70 mGy, while kidneys and spleen received up to 3.41 and 2.96 mGy, respectively. The highest ESDs were recorded in liver segments 2, 3, 4A, 4B, 5, and 6 as well as in the central regions of the kidneys, underscoring the influence of anatomical positioning and X-ray beam orientation. Overall, cathode-facing beams delivered up to 50% higher liver doses and twice the spleen dose compared to anode-facing beams.

Conclusions: This study confirms that radiation intensity is non-uniformly distributed across abdominal regions during X-ray imaging, with significant implications for patient safety. It demonstrates that different organs receive varying radiation doses, enhancing our understanding of organ-specific exposure during diagnostic procedures. These findings underscore the importance of optimizing imaging protocols to minimize unnecessary radiation exposure, particularly to more sensitive organs, ultimately improving patient care in diagnostic radiography.

## Introduction

The discovery of X-rays transformed medical care, allowing healthcare professionals to see inside the body and diagnose problems more accurately. Today, X-ray imaging is a vital tool in examining bones, organs, and tissues. While it does involve exposure to ionizing radiation, modern technology and careful use help keep the risks low, making the benefits more significant than the potential harm when used responsibly.

Abdominal radiography, which includes imaging of various organs within the abdominal cavity, such as the liver, spleen, gallbladder, and intestine, is an essential diagnostic procedure. One common form of abdominal radiography is kidney, ureter, and bladder (KUB) radiography, which targets the renal system. According to James and Kelly [1], abdominal radiography is typically performed with the patient in the supine position, where the X-ray beam passes from front to back in the anteroposterior (AP) projection. The clinical indications for abdominal radiographic examinations are normally grouped into suspected bowel obstruction, perforation, suspected foreign body, moderate-to-severe undifferentiated abdominal pain, and renal tract calculi follow-up [2].

Achieving uniform X-ray beam intensity is challenging due to factors like the heel effect (uneven absorption from the angled anode). The angled anode causes greater X-ray absorption on the heel side, reducing intensity compared to the cathode side, especially noticeable in large-field imaging [3]. Off-focus radiation further complicates uniformity as stray electrons produce low-energy, divergent X-rays that increase peripheral non-uniformity [4]. Moreover, beam divergence and the inverse square law naturally reduce intensity toward the periphery, while inconsistent filtration can introduce additional variations in beam hardening [5,6]. Collimation, though essential for reducing scatter, creates penumbral blurring at field edges due to the finite focal spot size [7]. Additionally, prolonged tube use leads to anode degradation, resulting in uneven X-ray production and localized intensity variations [8]. This variation in radiation intensity can result in uneven radiation exposure across the body, particularly for organs positioned at different points relative to the origin of the X-ray beam [9].

The entrance surface dose (ESD) is a critical dosimetric quantity in radiography that represents the amount of radiation absorbed by the skin at the point where the X-ray beam enters the patient's body. It is typically measured in grays (Gy) and serves as an important indicator of the radiation exposure that a patient receives during diagnostic imaging procedures, particularly X-ray examinations. ESD is essential for assessing patient safety and optimizing radiation protection protocols in the clinical setting [10-12].

The significance of ESD extends beyond mere measurement, as it plays a vital role in radiation protection and risk assessment. High ESD values indicate an increased risk of radiation-induced effects, particularly in sensitive populations such as children and pregnant women [13]. For instance, pediatric patients are more radiosensitive because of their rapidly dividing cells, making it crucial to monitor and minimize ESD during radiographic examinations [13,14]. Studies have shown that optimizing exposure parameters can significantly reduce ESD while maintaining diagnostic image quality, thereby enhancing patient safety [14-16].

Understanding the factors that influence ESD is essential for radiologists and radiographers in clinical practice. The key parameters affecting ESD include kilovolt peak (kVp), milliampere-seconds (mAs), source-to-image receptor distance (SID), and field size. Higher kVp settings generally result in lower ESD due to increased beam penetration, whereas higher mAs values lead to increased ESD because of the greater number of X-ray photons being produced [14,15,17]. Additionally, increasing the SID can reduce the ESD, as the intensity of radiation decreases with distance from the source [15,16]. Therefore, careful consideration of these parameters is necessary to achieve a balance between an adequate image quality and minimal radiation exposure.

The anode heel effect is a well-known phenomenon in radiography resulting from the angled anode design of X-ray tubes, leading to greater X-ray intensity on the cathode side and decreased intensity on the anode side [18-20]. This intensity variation can result in significant differences in radiation dose-up to 45%-to organs depending on their positioning relative to the beam, which is critical in procedures like lumbar spine or pediatric radiography [9,19,21]. It also affects image quality, especially on the anode side, potentially compromising diagnostic accuracy [22,23]. Radiographers can use this effect to optimize both image quality and patient safety by strategic positioning and shielding, and technologies like cone beam computed tomography (CBCT) further emphasize the need to account for this effect in dose planning and image optimization [24,25].

Despite advancements in X-ray imaging technology, accurately mapping and quantifying the radiation dose distribution to specific organs during abdominal radiography remains a significant challenge. Current organ dose estimation methods in X-ray diagnostic imaging frequently rely on standardized phantoms and generalized conversion coefficients, which fail to capture individual patient anatomy or specific exposure parameters. Such simplified approaches, often based on lookup tables or Monte Carlo simulations, can underestimate or overestimate organ doses, leading to inaccurate risk assessments and suboptimal radiation protection strategies [26]. As diagnostic imaging use grows and patients accrue cumulative exposure, the lack of precise, organ-specific dose information undermines efforts to balance diagnostic efficacy with safety. Although voxel-based Monte Carlo techniques offer more patient-specific dosimetry, their high computational demands have limited widespread clinical adoption [27]. Moreover, the absence of standardized methods for organ dose mapping that account for patient variability impedes the development of comprehensive radiation protection guidelines.

This study aimed to bridge the gap in understanding organ-specific radiation dose distribution by employing an innovative dose mapping approach using a Kyoto Kagaku abdominal phantom to measure ESD to corresponding abdominal organs. Specifically, investigating the radiation exposure of abdominal organs and examining the effects of non-uniform radiation intensity within the primary beam can lead to significant dose variations across different organs. By providing more accurate insights into radiation exposure risks and optimizing dose distribution, the study has the potential to enhance clinical practice, improve patient safety, and advance personalized medicine in diagnostic radiology.

## Materials and methods

**Figure 1:**
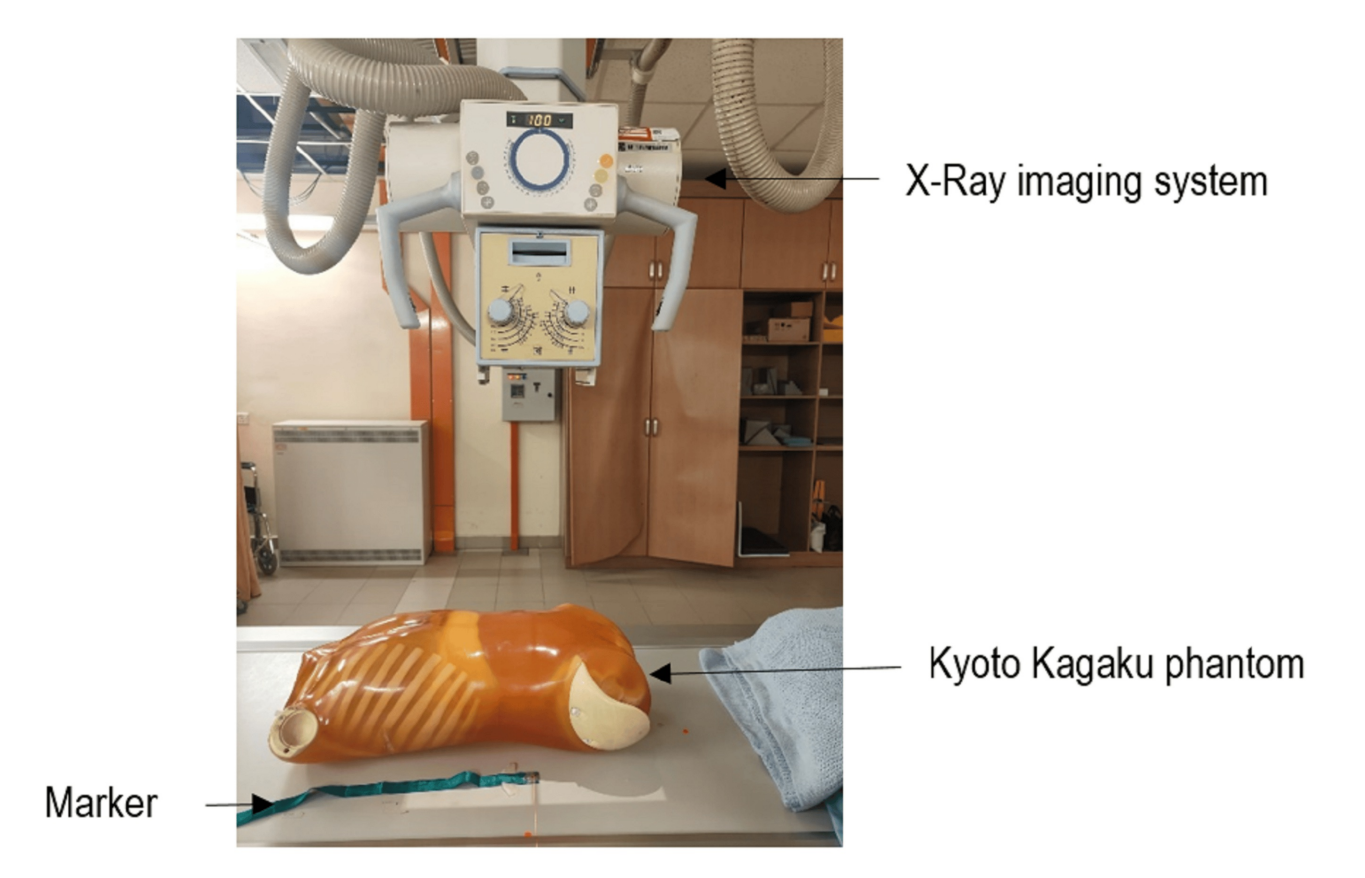
Experimental setup for measuring entrance skin dose (ESD) to abdominal organs (liver, kidneys, and spleen) during anteroposterior (AP) supine abdominal radiography. Created by the authors

This study used a Siemens Healthineers Multix Top X-ray system (Erlangen, Germany) and the Kyoto Kagaku PBU-50 whole-body phantom (torso) (Kyoto, Japan), which closely mimics human anatomy for accurate imaging and dosimetry. Optically stimulated luminescence dosimeters (OSLDs, Landauer nanoDot, Glenwood, IL, US) were employed for ESD measurement due to their high sensitivity (<1% precision) [28]. The microStar Landauer portable OSLD reader (with inLight software v5.0) was used to read OSLDs. OSLDs were read before the exposures to record their individual background readings, so they can be deducted from the measured dose after the exposure to get the true reading. The phantom was positioned on the table Bucky for abdominal AP supine projections (Figure 1).

**Figure 2:**
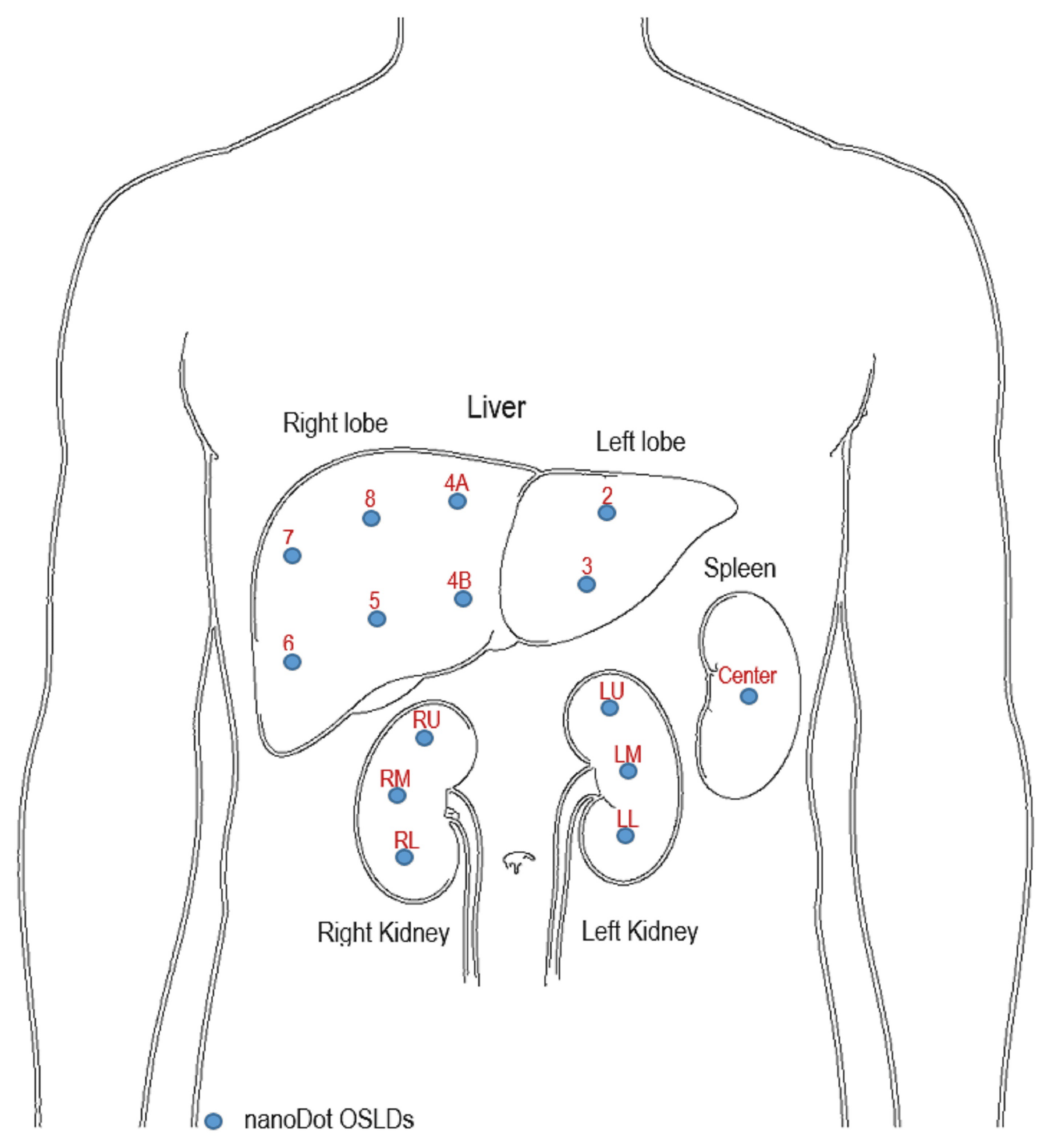
Anatomical segmentation of the liver, kidneys, and spleen. The liver is divided into eight segments: 2, 3, 4A, 4B, 5, 6, 7, and 8. Each kidney is divided into three regions: the right kidney into right upper (RU), right middle (RM), and right lower (RL) and the left kidney into left upper (LU), left middle (LM), and left lower (LL). The spleen is considered a single region in dose calculation. The nanoDot OSLDs positioned on specific regions of each organ to measure organ-specific ESD. Created by the authors OSLD: optically stimulated luminescence dosimeter; ESD: entrance surface dose

Figure 2 shows the traced representation of organs of interest including the liver, kidneys, and spleen. The liver is anatomically divided into eight segments: 2, 3, 4A, 4B, 5, 6, 7, and 8. Each kidney is divided into three regions: the right kidney into right upper (RU), right middle (RM), and right lower (RL) and the left kidney into left upper (LU), left middle (LM), and left lower (LL). The spleen is considered a single region in dose calculation. Due to its anatomical position at the periphery of the X-ray beam, it is assumed that there is minimal variation in dose distribution across the spleen. The nanoDot OSLDs were strategically placed on the traced organ sections as depicted in Figure 2 for precise ESD measurement. Exposure parameters included tube voltages of 64.5, 70, and 75 kVp and tube currents of 15, 20, and 25 mAs, respectively. The selected exposure factors were chosen based on their routine application in diagnostic abdominal radiography practice. A fixed SID of 100 cm was used, and the automatic exposure control (AEC) was turned off. Further details are provided in Table 1. To assess the anode heel effect on organ doses, the phantom was repositioned from the anode side to the cathode side. The procedure was repeated with varying exposure parameters (kVp and mAs) to evaluate the dose variations when the X-ray beam from the cathode side was directed toward the phantom.

**Table 1:** Parameters used for the study.

Parameters	Details
Exposure factors	64.5 kVp and 15 mAs, 70 kVp and 20 mAs, 75 kVp and 25 mAs
Imaging receptor (IR) size (cm)	35 x 45, portrait
Source-to-image distance (cm)	100
Focal spot	Broad focal spot
Automatic exposure control (AEC)	No
Central ray	Perpendicular to the center of IR, at the level of the iliac crest
Collimation	14 x 17 inches, field of view on all four sides to the anatomy of interest

Statistical analysis

Data were analyzed using International Business Machines (IBM), Statistical Package for the Social Sciences (SPSS) version 28 (IBM Corp., Armonk, NY, US) to assess significant differences in mean ESD across various exposure settings and beam orientations (anode- and cathode-facing) directed at the phantom. A p-value of <0.05 was considered statistically significant.

## Results

ESD measurements for abdominal organs using an anode-oriented X-ray beam

**Figure 3:**
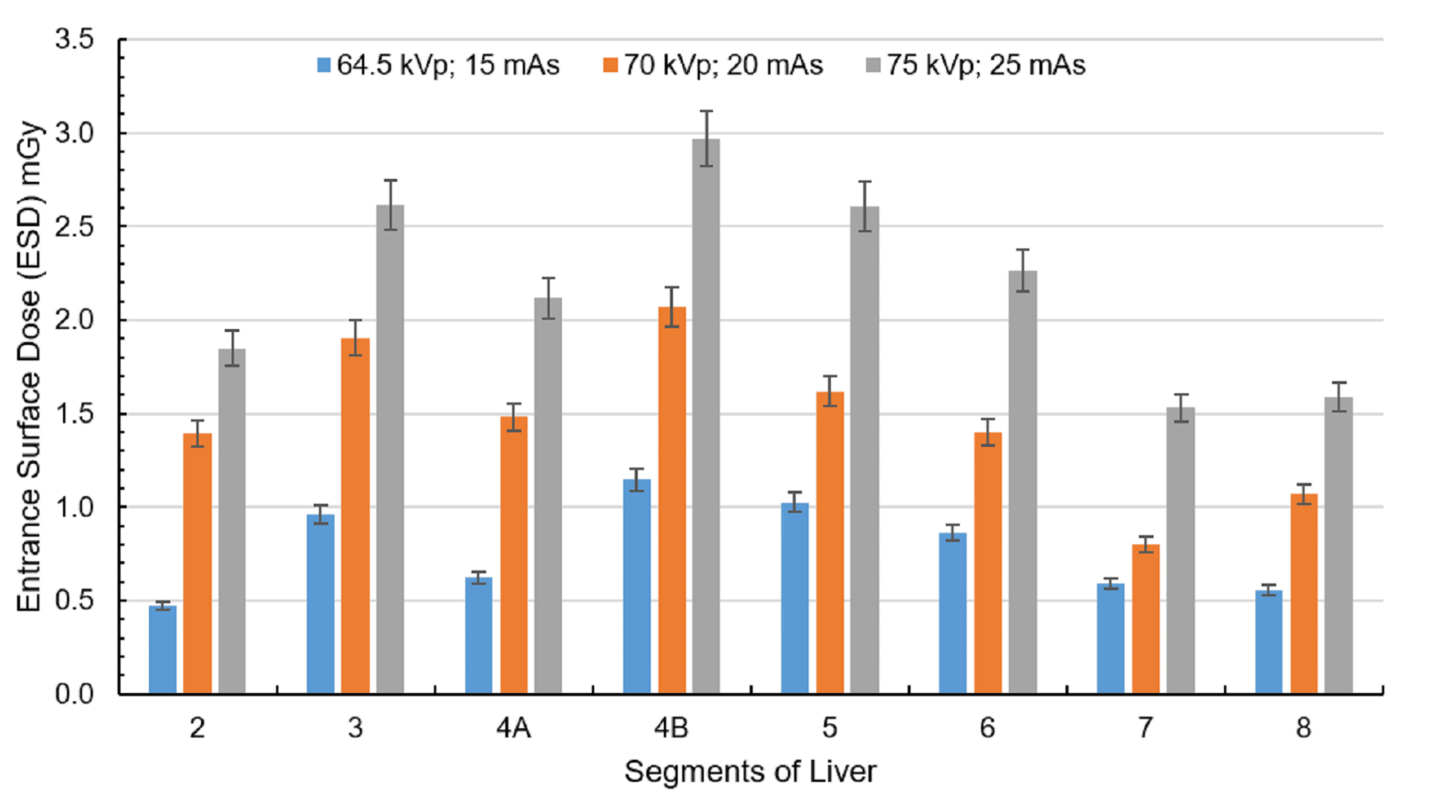
ESD corresponding to the liver at exposure settings of 64.5 kVp, 15 mAs; 70 kVp, 20 mAs; and 75 kVp, 25 mAs with the anode oriented toward the phantom. The liver is divided into eight segments: 2, 3, 4A, 4B, 5, 6, 7, and 8. Created by the authors

Figure 3 shows the ESD to the liver, measured using nanoDot OSLDs, increased with higher exposure factors (kVp and mAs). At the lowest exposure setting (64.5 kVp, 15 mAs), doses ranged from 0.47 to 1.14 mGy, with peripheral regions receiving lower doses and central zones showing higher values. When exposure increased to 70 kVp and 20 mAs, liver doses rose, particularly in regions 2, 3, 4A, 4B, 5, and 6. At the highest exposure setting (75 kVp, 25 mAs), the liver dose reached 2.97 mGy, with regions 2, 3, 4A, 4B, 5, and 6 exhibiting the highest values.

**Figure 4:**
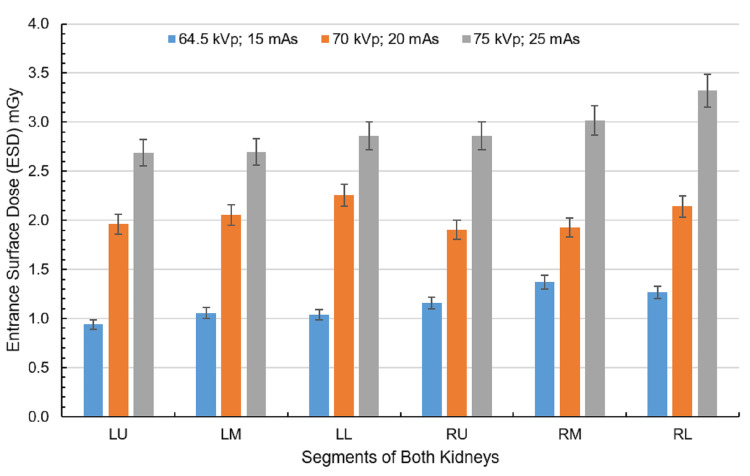
ESD corresponding to the kidneys at exposure settings of 64.5 kVp, 15 mAs; 70 kVp, 20 mAs; and 75 kVp, 25 mAs with the anode oriented toward the phantom. Each kidney is divided into three regions: the left kidney into left upper (LU), left middle (LM), and left lower (LL) and the right kidney into right upper (RU), right middle (RM), and right lower (RL). Created by the authors

The kidneys demonstrated a dose increase with increasing exposure factors as shown in Figure 4. At 64.5 kVp and 15 mAs, doses ranged from 0.93 to 1.37 mGy, with the left kidney’s upper (LU) and lower (LL) regions receiving the lowest doses, while the right kidney (RU, RL) showed higher values. With exposure factor (70 kVp, 20 mAs), kidney doses increased, with left-sided regions generally receiving higher doses than the right. At the highest exposure (75 kVp, 25 mAs), the RL kidney region received the maximum dose of 3.32 mGy.

**Figure 5:**
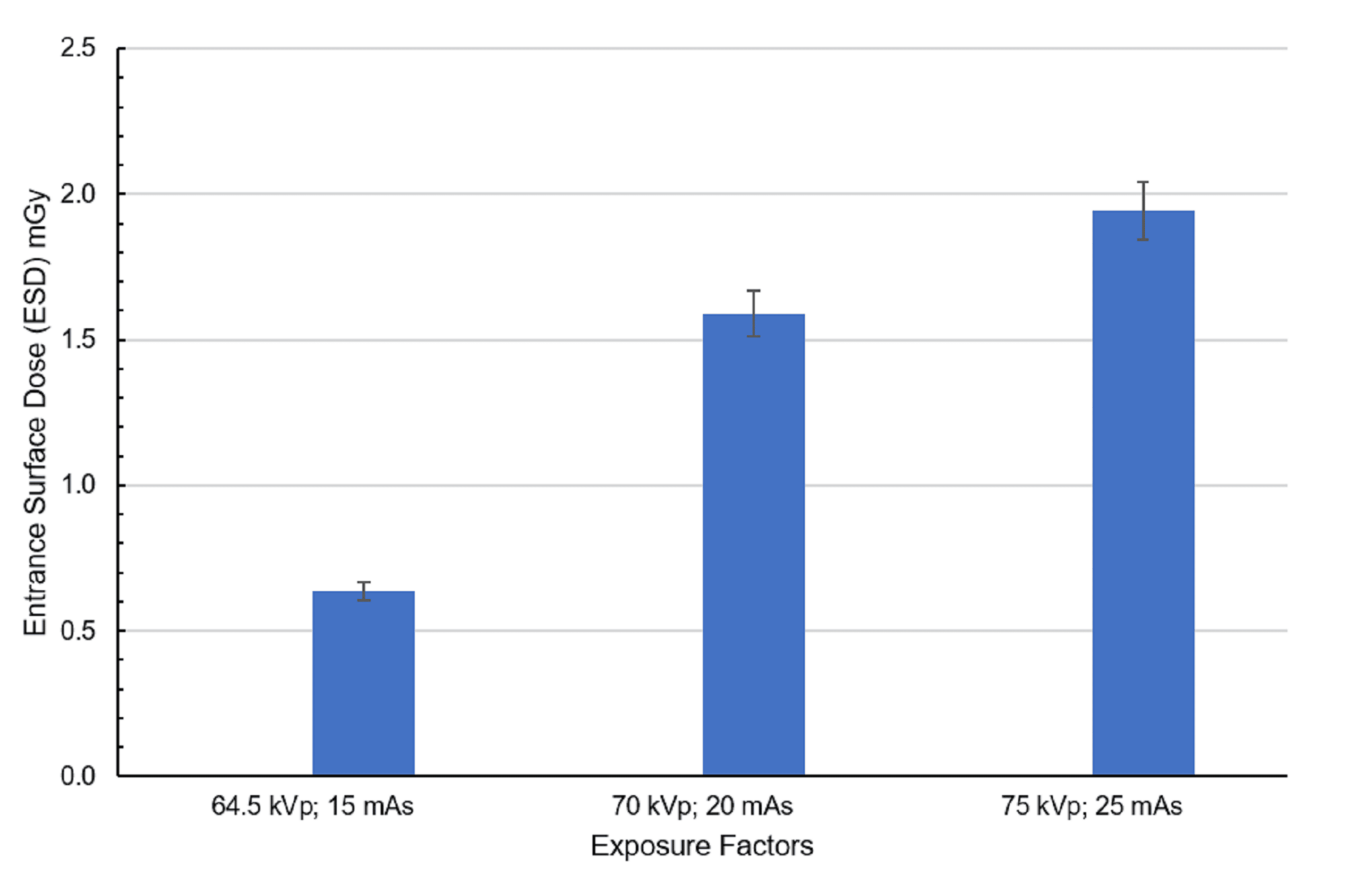
ESD corresponding to the spleen at exposure settings of 64.5 kVp, 15 mAs; 70 kVp, 20 mAs; and 75 kVp, 25 mAs with the anode oriented toward the phantom. The spleen is considered a single region in dose calculation. Created by the authors

The spleen, positioned more posteriorly and laterally, received the lowest dose (0.63 mGy) at the lowest exposure setting (64.5 kVp, 15 mAs) as shown in Figure 5. With increased exposure (70 kVp, 20 mAs), the dose more than doubled to 1.59 mGy. At the highest exposure setting (75 kVp, 25 mAs), the spleen reached its peak measured dose of 1.94 mGy, showing a consistent dose escalation in response to higher kVp and mAs values.

ESD measurements for abdominal organs using cathode-oriented X-ray beam

**Figure 6:**
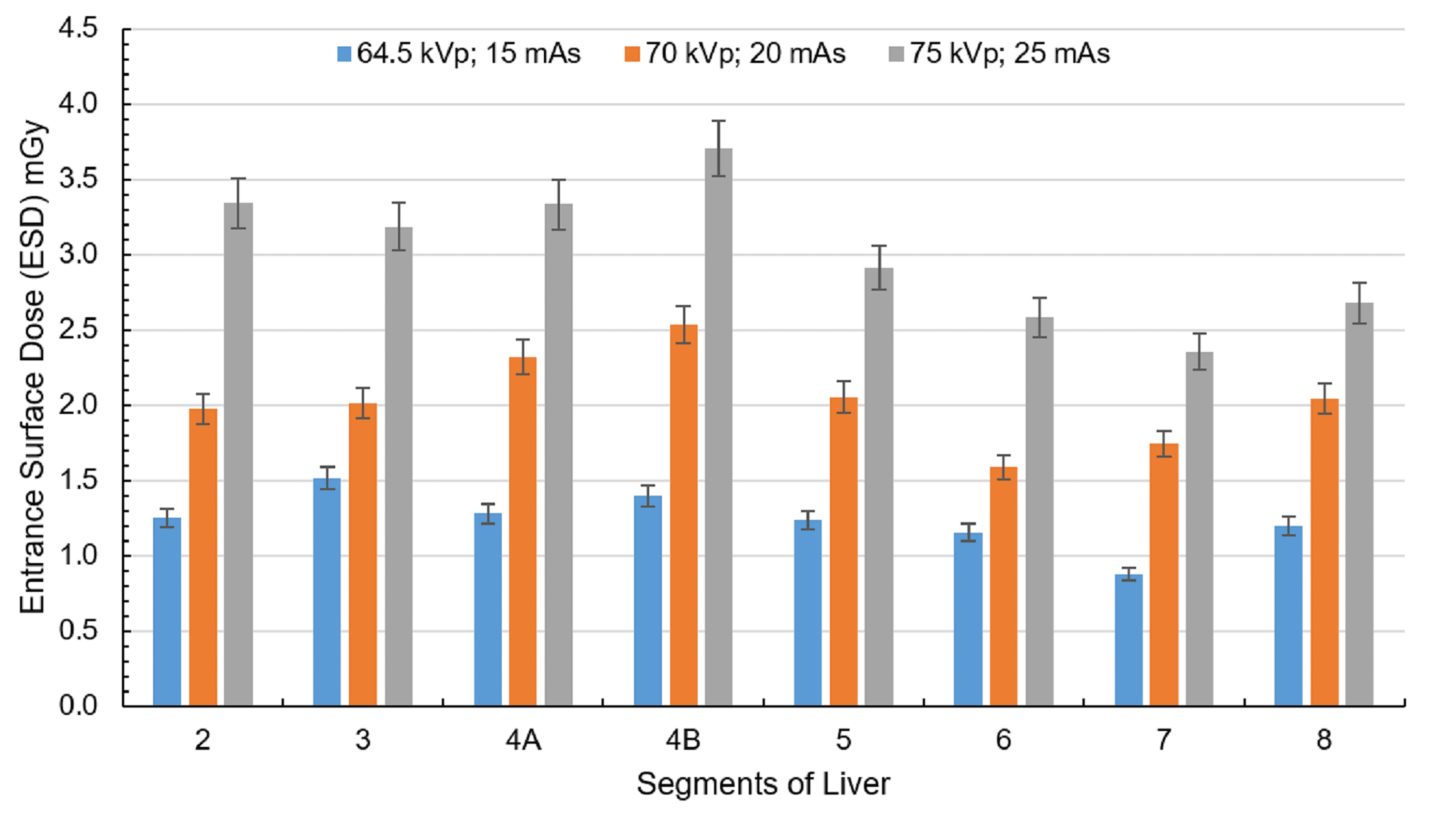
ESD corresponding to the liver at exposure settings of 64.5 kVp, 15 mAs; 70 kVp, 20 mAs; and 75 kVp, 25 mAs with the cathode oriented toward the phantom. The liver is divided into eight segments: 2, 3, 4A, 4B, 5, 6, 7, and 8. Created by the authors

Under the lowest exposure setting (64.5 kVp and 15 mAs), the liver exhibited radiation doses ranging from 0.88 to 1.51 mGy, with regions 6 and 7 receiving the lowest doses and regions 3 and 4B recording the highest, as shown in Figure 6. At the exposure setting of 70 kVp and 20 mAs, the overall liver dose increased, with regions 4A and 4B continuing to show elevated doses. Under the highest exposure (75 kVp and 25 mAs), the liver dose peaked, reaching a maximum of 3.70 mGy in region 4B, maintaining the trend of higher central doses observed in previous settings.

**Figure 7:**
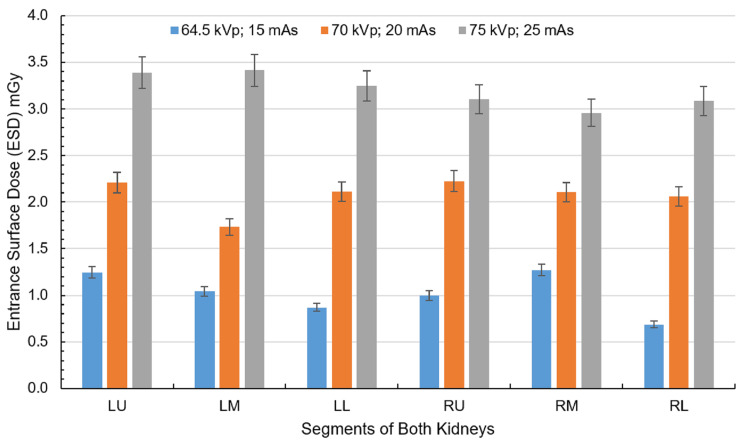
ESD corresponding to the kidneys at exposure settings of 64.5 kVp, 15 mAs; 70 kVp, 20 mAs; and 75 kVp, 25 mAs with the cathode oriented toward the phantom. Each kidney is divided into three regions: the left kidney into left upper (LU), left middle (LM), and left lower (LL) and the right kidney into right upper (RU), right middle (RM), and right lower (RL). Created by the authors

At 64.5 kVp and 15 mAs, kidney doses ranged from 0.68 to 1.27 mGy, with the LL and RL regions receiving the lowest doses and the LU and RM regions experiencing higher exposures, as shown in Figure 7. With increased exposure to 70 kVp and 20 mAs, kidney doses rose to a range of 0.94 to 1.37 mGy, with the LU and LL regions registering the lowest doses and the RM and LM regions receiving comparatively higher doses. At the highest setting (75 kVp and 25 mAs), kidney doses peaked, reaching a maximum of 3.41 mGy in the LM region and a secondary peak of 3.38 mGy in the LU region, indicating significant dose increases in the central and lower segments.

**Figure 8:**
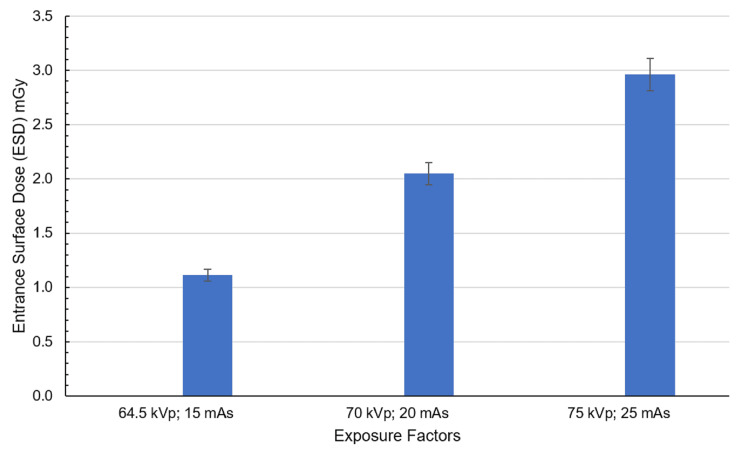
ESD corresponding to the spleen at exposure settings of 64.5 kVp, 15 mAs; 70 kVp, 20 mAs; and 75 kVp, 25 mAs with the cathode oriented toward the phantom. The spleen is considered a single region in dose calculation. Created by the authors

The spleen received a dose of 1.11 mGy at the lowest exposure setting (64.5 kVp and 15 mAs), representing the minimum among all conditions as shown in Figure 8. At the exposure setting of 70 kVp and 20 mAs, the spleen dose doubled to 2.04 mGy. Under the highest exposure (75 kVp and 25 mAs), the spleen received its peak dose of 2.96 mGy, demonstrating a consistent and proportional increase with rising exposure parameters.

Comparison of ESD for abdominal organs: anode- vs. cathode-oriented X-ray beams

The liver exhibited notable differences in ESD depending on X-ray beam orientation. Under anode-oriented beams, ESD ranged from 0.47 to 1.14 mGy at the lowest exposure (64.5 kVp, 15 mAs), with central regions (e.g., segments 4A and 4B) receiving higher doses than peripheral areas. Increasing exposure factors led to the increase in ESD, peaking at 2.97 mGy (75 kVp, 25 mAs). In contrast, cathode-oriented beams delivered consistently higher ESDs across all settings, starting at 0.88-1.51 mGy (64.5 kVp, 15 mAs) and reaching a maximum of 3.70 mGy (75 kVp, 25 mAs). Central liver regions (particularly 4B) showed the most pronounced increase under cathode orientation.

The kidneys demonstrated distinct dose distribution patterns based on beam orientation. With anode-oriented beams, the right kidney (RU, RL) received higher doses at low exposures (0.93-1.37 mGy at 64.5 kVp, 15 mAs), while increasing exposure shifted the dose toward the left kidney (LU, LL). The highest anode-side ESD was 3.32 mGy in the RL region. Conversely, cathode-oriented beams increased the left kidney, particularly the medial (LM, LU) regions, with doses peaking at 3.41 mGy (75 kVp, 25 mAs).

The spleen, located posteriorly and laterally, showed a notable difference between beam orientations. Under anode-oriented beams, doses started at 0.63 mGy (64.5 kVp, 15 mAs) and peaked at 1.94 mGy (75 kVp, 25 mAs). However, cathode-oriented beams delivered double the dose at low exposures (1.11 mGy) and reached 2.96 mGy at maximum settings, an ~50% increase compared to anode orientation.

Overall, cathode-oriented beams resulted in higher ESD values for all three organs compared to anode-oriented beams. The liver and spleen exhibited the most pronounced increases, with central liver regions and the spleen receiving up to 25%-50% higher doses under cathode orientation. The kidneys showed a shift in dose distribution, with anode beams increasing the right kidney and cathode beams increasing exposure to the left kidney.

## Discussion

The findings from this study demonstrate that X-ray beam orientation-whether anode- or cathode-oriented-significantly influences radiation dose distribution across abdominal organs, with notable variations observed in the liver, kidneys, and spleen. The cathode-oriented beam consistently delivered higher doses to all measured organs compared to the anode-oriented configuration, a phenomenon likely attributable to the anode heel effect, wherein the cathode side of the X-ray beam exhibits greater intensity due to reduced self-absorption. This effect was particularly evident in the spleen, which received nearly double the dose in cathode-facing exposures (1.11 mGy vs. 0.63 mGy at 64.5 kVp and 15 mAs), suggesting that posteriorly positioned organs are more susceptible to radiation when the cathode is oriented toward the phantom.

Furthermore, the kidneys exhibited a directional dependence, with the anode-oriented beam favoring higher right kidney doses (peaking at 3.32 mGy in the RL region) and the cathode-oriented beam increasing left kidney exposure (peaking at 3.41 mGy in the LM region). This asymmetry underscores the impact of beam alignment on organ-specific radiation burden, possibly due to differences in tissue attenuation and anatomical positioning relative to the primary beam path. The liver also displayed regional variations, with central segments (e.g., Couinaud segments 4A and 4B) consistently receiving higher doses in both orientations, though cathode-facing exposures exacerbated this trend, reaching a maximum of 3.70 mGy compared to 2.97 mGy in anode-facing setups.

These results have important clinical implications, particularly in optimizing radiographic techniques to minimize unnecessary radiation exposure. For instance, if reducing spleen or left kidney dose is a priority (e.g., in pediatric imaging or patients with pre-existing conditions), an anode-oriented beam may be preferable. Conversely, if right kidney sparing is desired, a cathode-facing approach might be more suitable. Future studies could explore additional variables, such as patient positioning and beam filtration, to further refine dose optimization strategies in abdominal radiography. Overall, this study highlights the necessity of considering beam orientation in clinical practice to achieve a balance between diagnostic efficacy and radiation safety.

The ESD in abdominal X-ray radiography is significantly influenced by various exposure parameters, including kVp, mAs, SID, and field size. Understanding how these parameters interact is crucial for optimizing patient safety and ensuring diagnostic efficacy. The kVp setting directly affects the energy of the X-ray beam, which in turn influences ESD. Higher kVp values result in more penetrating X-rays, which can reduce ESD while maintaining the image quality. For instance, Adejoh et al. [29] reported that increasing kVp from 60 to 90 can lead to a substantial decrease in ESD by approximately 60%, as noted in studies focusing on chest radiography. This principle also applies to abdominal radiography, where higher kVp settings can effectively lower ESD while still producing diagnostically useful images. However, it is essential to balance kVp with the specific anatomical characteristics of the patient because denser tissues may require a higher kVp to achieve adequate penetration [30,31].

The mAs setting, which determines the quantity of X-rays produced, is another critical factor affecting the ESD. Higher mAs values increase the number of generated X-ray photons, leading to a higher ESD. Conversely, reducing mAs can lower ESD but may compromise image quality if not carefully managed. Studies have shown that adjusting mAs according to patient-specific parameters such as body mass index (BMI) and age can help optimize ESD while maintaining diagnostic quality [31].

SID also plays a vital role in determining ESD. A shorter SID results in a higher dose to the skin owing to the inverse square law, which states that the radiation intensity decreases with the square of the distance from the source. Therefore, increasing the SID can effectively reduce ESD. This principle is particularly relevant in abdominal radiography, where positioning the X-ray tube further away from the patient can minimize skin exposure while still achieving adequate image quality [30,31].

Field size is another parameter that can influence ESD. Collimating the X-ray beam to the area of interest not only improves image quality by reducing scattered radiation but also minimizes the radiation dose to the surrounding tissues. Studies have demonstrated that careful collimation can significantly reduce ESD, emphasizing the need for radiographers to routinely employ this technique [30,31].

Moreover, the type of imaging system used can also affect the ESD. The transition from screen-film systems to digital radiography (DR) has necessitated a re-evaluation of diagnostic reference levels (DRLs) for various X-ray examinations, including abdominal radiography. Research indicates that DR systems may require different exposure settings to achieve similar ESD levels compared with traditional systems, necessitating ongoing assessment and adjustment of exposure parameters [31,32].

In addition to these parameters, patient-specific factors, such as age, height, and weight, must be considered when determining ESD. For instance, pediatric patients are more sensitive to radiation, and adjustments in the exposure parameters are crucial for minimizing their risk [33,34]. Studies have shown that pediatric ESDs can vary significantly based on the exposure settings used, underscoring the importance of tailored approaches in this population [33,34].

The observed radiation dose distribution across the liver, kidneys, and spleen clearly demonstrates a dose-dependent relationship with increasing exposure parameters (kVp and mAs), highlighting the direct influence of X-ray energy and tube current-time product on organ radiation burden. At the lowest exposure setting (64.5 kVp, 15 mAs), all three organs received lower doses exhibiting minor heterogeneity in intra-organ distribution, reflecting anatomical orientation and tissue depth relative to the beam. Notably, the kidneys, especially the right side, showed higher doses, which may be attributed to their position in the primary beam path. As exposure settings increased to 70 kVp/20 mAs and further to 75 kVp/25 mAs, there was a consistent and substantial escalation in organ doses. Liver segments 3, 4A, 4B, and 5 consistently received higher doses, indicating preferential alignment with the radiation beam and reduced shielding by surrounding tissues. The kidneys, particularly the right lower region, exhibited a notable increase in radiation dose. Although the spleen received comparatively lower doses due to its posterior anatomical position, a consistent upward trend in ESD levels was observed.

Overall, the interplay between kVp, mAs, SID, and field size is complex, and optimizing these parameters is essential for minimizing ESD while ensuring diagnostic quality in abdominal radiography. Accurate measurements and assessments of ESD are crucial for ensuring patient safety, optimizing imaging protocols, and minimizing risks associated with radiation exposure. Consistent monitoring and strict adherence to established guidelines help minimize the potential adverse effects of radiation while preserving the effectiveness of diagnostic imaging procedures. Furthermore, continuous education and training for radiographers on these factors can significantly enhance patient safety and improve imaging outcomes.

Limitations of the study

This study has a few important limitations. First, it used a physical phantom rather than real patients. While phantoms allow for consistent measurements, they do not fully represent human anatomy, tissue differences, or organ motion, which may affect how the findings apply in clinical practice. Second, the study was conducted using only one X-ray system. Since imaging equipment varies in performance and settings, the results may not be generalizable to other machines. Future studies should consider using multiple systems and more realistic models to better reflect real-world conditions.

## Conclusions

In this study, we found that radiation doses to the liver, kidneys, and spleen increased consistently with higher X-ray exposure settings (kVp and mAs), regardless of whether the beam was anode- or cathode-oriented. The liver, particularly its central regions, received the highest doses, followed by the kidneys and spleen. Notably, cathode-oriented beams delivered more radiation to the organs than anode-oriented beams, likely due to the heel effect, which intensifies the beam on the cathode side. These findings underscore the importance of optimized patient positioning during imaging. Placing radiosensitive organs away from the cathode side can help reduce unnecessary exposure. They also highlight the need for well-designed imaging protocols that incorporate appropriate exposure settings, precise collimation, and beam orientation to minimize radiation risk while maintaining diagnostic image quality. Future research should investigate additional exposure parameters, phantom orientations, and patient-specific models to further enhance dose optimization in abdominal radiography.
